# Reply to Hasenkrug et al., “Different Biological Activities of Specific Interferon Alpha Subtypes”

**DOI:** 10.1128/mSphere.00275-19

**Published:** 2019-07-24

**Authors:** Erika Schlaepfer, Audrey Fahrny, Maarja Gruenbach, Stefan P. Kuster, Viviana Simon, Gideon Schreiber, Roberto F. Speck

**Affiliations:** a Division of Infectious Diseases and Hospital Epidemiology, University Hospital of Zurich, Zurich, Switzerland; b Department of Microbiology, The Global Health and Emerging Pathogens Institute, Icahn School of Medicine at Mount Sinai, New York, New York, USA; c Department of Biomolecular Sciences, Weizmann Institute of Science, Rehovot, Israel

## REPLY

We appreciate the reply by Hasenkrug et al. The basic issue we addressed in our paper was whether the differences between the different interferon alpha (IFN-α) subtypes are quantitative or qualitative. In our paper, we noted that all of the IFN-α subtypes are able to inhibit HIV replication in various primary cell types to the same extent, depending upon the dose given (1). Furthermore, we presented a limited data set of IFN-stimulated genes (ISGs) which were quite similar irrespective of the distinct IFN-α subtype added to the cell culture. On the basis of these findings, we concluded that IFN-α subtypes do not induce different biological responses. In other words, the difference between IFN-α subtypes in relation to HIV replication inhibition is quantitative and not qualitative. Hasenkrug et al. came to a different conclusion in their work (2, 3), i.e., that distinct IFN-α subtypes, among others, IFN-α6, IFN-α8, and IFN-α14, have a more potent anti-HIV activity than other subtypes and that the differences are related to distinct biological activities. While they showed in Fig. 1 of their work that IFN-α14 had a higher level of potency (11-fold) than IFN-α2 (in line with its higher receptor binding affinity and antiviral activity against vesicular stomatitis virus [VSV; 4]), all other experiments were done at one IFN-α concentration. From that point, they concluded that the differences between IFN-α2 and IFN-α14 are qualitative, while they themselves showed that the differences are actually quantitative and are related to receptor binding affinity (3, 4).

IFN-α subtypes have distinct affinities to the IFN receptor (4), and there is a consensus that level of affinity determines the strength of signaling. Moll et al. also reported that differences in ISG levels were only seen at subsaturating levels (5). Thus, we are challenged when studying IFN-α subtypes in whether we should design experiments comparing IFN-α subtypes using equimolar doses or functional units and at what dose(s). Data generated with identical functional units of distinct IFN-α subtypes may rather reflect the higher potency of a given IFN-α subtype as assessed in a first round of experimentation for determining the functional units. In the end, we are convinced that we need stringent *in vitro* as well as *in vivo* dose-response investigations to compare IFN-α subtypes.

Furthermore, Lavender et al. argued in their paper that they had chosen a high dose of IFN-α2 to demonstrate the maximal efficacy that would be clinically achievable by IFN-α2 compared to the same unit dose of IFN-α14 (2). This argument is problematic. First, we do not know to what extent the same unit dose of IFN-α14 is tolerated in human or mice, as we lack such data. Second, and more important, the half-life of proteins in mice is much shorter than in humans; thus, the same (weight-adjusted) dose in mice would have a lesser effect than in humans, and higher weight-adjusted doses are therefore required in mice. At the least, it appears that the humanized mice showed good tolerance of the dose used by Lavender et al., but let us remember that IFNs are species specific and that humanized mice will most likely not be the model used to explore undesirable side-effects of IFNs.

We entirely agree that *in vitro* experiments must be followed by *in vivo* experiments. We highly appreciate the data presented by Lavender et al. (2). We also entirely agree that IFNs may act differently with cell type-specific responses in the microenvironment and that this can hardly be recapitulated by *in vitro* experiments. Furthermore, pharmacological doses of IFNs administered exogenously may have biological activities that are distinct from those of IFNs released in the microenvironment. From our point of view, the chapter on IFN subtypes (and, in particular, whether they have distinct biological activities) is not closed.
